# A Bayesian hierarchical mixture model for extracting process features in a newly developed cloud-based writing platform

**DOI:** 10.3389/fpsyg.2026.1792834

**Published:** 2026-05-28

**Authors:** Tingxuan Li, Shengwei An

**Affiliations:** 1 School of Education, Shanghai Jiao Tong University, Shanghai, China; 2 Department of Computer Science, Virginia Polytechnic Institute and State University, Blacksburg, VA, United States

**Keywords:** cloud-based platform, fully Bayesian framework, writing assessment, writing competency, writing process

## Abstract

In a computer-based writing platform, keystroke log data can document a student’s writing process (e.g., typing behaviors). A set of tools has been developed to collect the resulting log data. However, these tools originate from decades ago, and none of them has been adapted for use with cloud servers. In this research, we describe a cloud-based writing platform that we have developed, *Clourite*. It offers a series of benefits including improved scalability and automatic data collection. It is easy to use and requires only a few mouse clicks without installing any software. Moreover, with a sample of 309 college students, we collected empirical keystroke log data through *Clourite* and extracted writing process features using a Bayesian hierarchical mixture model. The non-hierarchical mixture model may enlarge the standard error when the number of pause events in that component is small, as shown in our previous work. In contrast, the proposed hierarchical model in a fully Bayesian framework has the advantage that essays are considered similar data units, meaning the model can *borrow strength* from longer essays to enhance the efficiency of estimation for shorter essays. We identified a distinct writing process pattern differentiating stronger and weaker writers, along with the association between process features and writing quality measures (i.e., human scores). These findings underscore the potential of keystroke logging analysis for characterizing student writing processes.

## 1 Introduction

Writing demands substantially more conscious effort than other literacy skills (e.g., reading). The writing process itself can be understood as a dynamic system (van den Bergh et al., 2016), comprised of interrelated cognitive and cognitive-linguistic activities including idea generation, organization, goal setting, formulation, rereading, evaluation, and revision. Research has consistently emphasized the importance of these mental operations. Hayes and Flower (1980), for instance, observed that skilled writing entails the monitoring of one’s own composing processes. Furthermore, studies have shown that the effective orchestration of these activities is closely linked to text quality (Barkaoui, 2019). The appeal of computer-based tools lies in their ability to automatically and non-intrusively record these processes through keystroke logging (Swar and Mohsen, 2023). By capturing a series of time-stamped actions, keystroke logging enables researchers to examine the behavioral aspects of writing (Leijten and Van Waes, 2013). Consequently, these data have provided insights into the writing processes (e.g., Alves et al., 2007; Olive et al., 2009; van Waes et al., 2014).

### 1.1 Research Goal 1: proposing a cloud-based writing platform-*Clourite*

After reviewing the literature, we discovered that a cloud-based writing platform is still lacking. Hence, we present a cloud-based writing platform that we have developed-*Clourite*. Although several Google doc extensions such as *DocuViz* and *Draftback*, along with browser-based extensions such as *Process Feedback*, have been developed to document writing processes or visualize editing histories, these tools still present certain limitations. Firstly, these tools rely on public cloud services, that indicates that stable internet access is required for their operation. In contrast, *Clourite*, supports both public cloud deployment and private cloud deployment. This flexible architecture allows the keystroke logging system to function even in environments where external internet access is restricted. For example, in some school computer laboratories, external network connections are not permitted. In such cases, Clourite can be deployed on a private cloud within the local network, making it a practical and feasible solution for writing process research and classroom use. Plus, with private cloud deployment, data are relatively more secure and private. Secondly, DocuViz and Draftback that rely on Google services face accessibility limitations in certain countries (regions) due to regulatory policies. Thirdly, researchers who conduct statistical analysis for keystroke log data collected during writing tend to work on the unit measured by millisecond. The precise timestamp is very important for statisticians (e.g., quantitative analysts). However, the existing tools are not designed to record the precise timestamps. In contrast, Clourite is designed to include these functionalities: (1) automatic recording, (2) precise timestamps, (3) continuous logging of writing actions. Namely, the millisecond measurement requires continuous, high-precision logging of every writing action, that are provided by Clourite.

Other than the tools mentioned above, some desktop applications (e.g., Inputlog, Leijten and Van Waes, 2013; Strömqvist and Karlsson, 2001) do possess compelling functionalities. For example, they provide (1) automatic recording, (2) precise timestamps, and (3) continuous logging of writing actions. But these tools are lack of cloud servers. In contrast, Clourite is a cloud-based infrastructure and a comprehensive ecosystem that integrates both learning activities (e.g., homework writing assignment) and summative assessment occasions (e.g., final writing exams) in a coherent way. So, it offers additional research affordances, that is, the cloud server solves specific challenges that these existing keystroke logging tools are unable to solve.

The first challenge is about the deployment and installation. The existing desktop tools (e.g., Inputlog) must be installed on individual computers in order to collect keystroke log data. As a result, data collection requires preparing each computer device in advance. This can make deployment difficult in settings such as: (1) institutions with restricted software installation and (2) distance learning (e.g., MOOCs or other large-scale remote learning courses). Plus, whenever there is a new update, the users must reinstall the software.

To solve this challenge, Clourite merely requires a user to complete a few of simple mouse clicks without installing any software on each machine. Namely, the cloud solution widens the usage and make it a practical and feasible solution for writing process research and classroom use. The screenshots that are provided in the next section illustrate how users can (1) assign writing task, (2) grade essay, (3) watch replay, (4) download process data and so on. The 3-min tutorial video link has been uploaded for general users to master the usage (https://github.com/TingxuanL/writing/wiki). Clourite can be accessed directly through a web browser, allowing researchers and practitioners (e.g., classroom teachers) to administer writing tasks to about 2000 students at the same time and collect synchronized process data.

The second challenge is about limited support for mobile and tablet devices. The existing desktop tools merely work on the Windows system. Although recently, an add-on for LibreOffice, so called ‘Inputlog-LibreOffice’, is developed (Buschenhenke et al., 2026). This add-on function allows data collection through not only Windows system, but also macOS, Linux, ChromeOS systems. However, the data collected here are still a large volume of raw information. In order to parse (classify) the raw information into meaningful structured writing process data (e.g., pause duration and location), the users still need to download Inputlog software to further acquire the structured datasets, meaning the users must download Inputlog that merely works on Windows system. Plus, LibreOffice also must be installed in order to use this ‘Inputlog-LibreOffice’ (Buschenhenke et al., 2026).

To solve this challenge, Clourite contains the cloud infrastructure that may substantively enhance the scalability. Namely, Clourite can be accessed through a web browser on both desktop operating systems (e.g., Windows, macOS, Linux) and even mobile operating systems (e.g., Android and iOS). In other words, students can use their own devices to complete the writing in class. From a scalability perspective, cross-device compatibility supports bring-your-own-device (BYOD) learning environments, where students can engage learning activities using their personal laptops, tablets, or smartphones (e.g., Chilton et al., 2025).

The third challenge concerns the fragmented data management. In the existing desktop tools (e.g., Inputlog), data are stored locally on individual computers and must be exported and transferred manually. For example, researchers need to manually collect and merge log files; teachers (or researchers) must manually release (probably verbally inform) the writing prompt to students (or participants); researchers must manually record the duration of the writing session.

To solve this challenge, Clourite employs a cloud-based infrastructure that facilitates virtual data management for both small-scale laboratory studies and large-scale classroom research. Because the platform is hosted on a cloud server, multiple users can access the system simultaneously without the need for manual transfer of any files or data. In Clourite, once a student submits an essay on the *student interface*, the essay text immediately appears on the *teacher’s interface*. Teachers can then grade the essays and assign holistic scores within the same platform. Subsequently, researchers can download both the structured writing process data (e.g., pause locations and durations) and the human scores (i.e., holistic ratings provided by teachers) in a single dataset file through the *admin interface*. The cloud-based architecture also allows flexible configuration and user roles. For example, through the *teacher interface*, teachers can pre-define the writing prompt and set a time limit for the writing task (e.g., 30 min). When students sign in to the *student interface*, the platform automatically tracks the duration of the writing session, eliminating the need for manual time recording. Once the time limit is reached, the system automatically terminates the writing session and saves all writing data.

The fourth challenge is about the difficulty integrating with smart learning environments. The existing desktop tools are designed primarily as standalone desktop applications, as mentioned above, that is, data must be exported and uploaded manually. To solve this challenge, the writing platform, Clourite, enhance the extensibility by introducing several architectural innovations that expand the methodological possibilities for writing process research. (1) Clourite adopts a *modular architecture*, in which different functional elements are clearly encapsulated. This design allows new modules to be added without modifying the core system, making it easier to extend the platform with additional research functionalities, such as new writing analytics, feedback mechanisms, or data visualization tools. The modular structure therefore facilitates the continuous development of the platform as research needs evolve. (2) Clourite is designed to operate on public or private cloud infrastructures, which makes it naturally compatible with emerging AI technologies, including large language models (LLMs). Clourite can connect to publicly available LLM services or deploy locally hosted LLMs within a private cloud environment, depending on institutional requirements for data security and privacy. (3) This architecture enables the integration of AI-driven agent systems that can support both instructional and research purposes. For example, such agents can automatically generate writing prompts and perform automated analyses of students’ writing behaviors. These capabilities open new possibilities for integrating writing process research with intelligent technologies and AI-supported platform.

### 1.2 Research Goal 2: proposing a Bayesian hierarchical mixture model

To evaluate an essay, advances in technology do not only provide score but also detailed information about how students compose their texts. (Note that the *score* is a generic term here. It is not only generated from a summative assessment occasion, but also generated from the evaluation of a daily writing assignment). Students may differ in their writing behaviors, such as whether they write continuously, pause frequently, or engage in planning and revision. Such process data can eventually help teachers identify students’ difficulties in fluency, planning, revision, and transcription. Summarizing these patterns at the individual or group level provides a valuable basis for instructional decision-making. Teachers can eventually use this information to model effective writing processes or highlight problematic behaviors. In order to achieve this, the researchers have pointed out, the initial attempt is to use statistical models as a starting point to explore whether the unique pattern is found in terms of writing process difference among students (e.g., Zhu and Gu, 2023; Guo et al., 2018).

Therefore, other than describing the innovative cloud-based writing platform, Clourite, in the present research, we also aim to (1) use Clourite to collect empirical process data and (2) extract process features using a Bayesian hierarchical mixture model. The hierarchical mixture models offer several potential benefits compared with their non-hierarchical alternatives. In mixture models, the standard error of the estimation for the parameters in a component depends on the number of events in that component. The non-hierarchical mixture model may enlarge the standard error when the number of events in that component is small, as shown in our previous work (Li, 2021). This is also the case when the magnitude on that mixing probability is small; consequently, the number of events is small. *Borrowing strength* is a well-known virtue in the fully Bayesian framework (Priebe, 1996; Mesquita et al., 2020). Under Bayesian hierarchical model’s core assumption, *exchangeability*, essays are viewed as similar data units. Shorter essays contain fewer events, which are most in need of improved inference. The model therefore borrows strength from other essays to enhance the efficiency of estimation for shorter essays.

In the context of the present research, once each student submits an essay online, the data possess a hierarchical structure; that is, keystroke log data are nested for each student. (Henceforth, we use “essay” and “student” interchangeably). By the standards of a Bayesian hierarchical mixture model, the parameters of each student are random variables drawn from prior distributions. All students’ mixture parameter estimates are shrunk towards the grand mean, which is an approach known as partial pooling (Gelman et al., 2013).

## 2 Related work

In this section, we reviewed the literature in three areas: (1) the cognitive models of writing, (2) the statistical models for quantifying keystroke log data, and (3) the existing keystroke logging writing tools.

### 2.1 Writing process from writing experts’ perspective

Writers purposively solve a set of complex rhetorical and linguistic problems of composition during text production. The fine-grained mental operations employed by writers include, for example, formulating plans, retrieving content from memory, organizing ideas, and converting linguistic strings to text (MacArthur and Graham, 2016). Chenoweth and Hayes (2001) developed a “four processes model”, in which the writing process contains (1) proposer, (2) translator, (3) reviser, and (4) transcriber subprocesses. Subsequently, Leijten et al. (2014) further refined the model by highlighting the role that the “search for content” plays. When planning or reviewing, students tend to search for what to write about, and how to elaborate it in a coherent way. Along this line, Latif (2021) further pointed out that it is necessary to explicate searching for content and monitoring in the cognitive model of writing. “Searching for content” is defined as identifying the ideational content needed for text production, by retrieving it from memory. “Monitoring” refers to the activity of adjusting the information retrieved from memory. These processes have been assumed to be guided by the central executive retrieving knowledge.

Deane et al. (2008) and Deane (2011) approximately categorized the cognitive activities that occur during writing into “low” and “high” processes. The underlying phenomenon is that a student (a writer) simultaneously coordinates multiple levels of cognitive skills, including the word level, sentence level, paragraph level, document level, and rhetorical-goal level. This approach reflected Kellogg (1996)’s idea that cognitive models of writing lower-and higher-level composition processes. Although effortful mental operations may occur in any order during text production, low and high processes compete for the limited resource of working memory.

### 2.2 Statistical models for quantifying keystroke log data

Keystroke log data can convey information about where a student has paused and for how long (measured in milliseconds) the student has paused during writing. Pause events are classified according to the linguistic context (i.e., text boundaries, Medimorec and Risko, 2017). For example, if a student paused when he/she was spelling a word, this event observation belongs to the “within word” linguistic context. If a student pauses between words, this observation belongs to the “between words” linguistic context. A single pause event is classified into only one applicable linguistic context. Existing studies have mainly employed two types of methods to quantify process data: (1) descriptive statistics or (2) data reduction techniques. Pause frequencies between words have been found meaningful across different writing tasks (Conijn et al., 2022). The underlying dimensionality of pause events data has typically been identified using principal component analysis (Baaijen et al., 2012; Zhang and Deane, 2015; Bennett et al., 2022).

In addition, several studies have employed data mining techniques. Guo et al. (2019) classified writing process data into various writing states, using a semi-Markov process. Owing to their large sample size was large (*N* = 2,500), these authors aimed to understand how students’ demographic groups (i.e., race, gender, and socioeconomic status) might affect transition probability in a semi-Markov model. Uto et al. (2020) adopted a Gaussian hidden Markov model (GHMM) to identify the multiple latent states during writing. These authors incorporated parameters were employed to express state appearance probabilities for each time interval about each student, and discovered that students with high and low writing competencies displayed different magnitudes on the ratios of states. Sinharay et al. (2019) used the data mining methods for classification and regression (DMMCR) model to predict human scores. In this case, the predictors were both product and process features. The product features were obtained using the e-rater in the AES. In contrast, the process features included many variables, such as the mean pause across a whole essay. Subsequently, Conijn et al. (2022) also used DMMCR with a different sample as a replication study, to invalidate Sinharay et al.’s findings. He et al. (2025) used process data from 4,309 essays to develop a deep learning model in order to enhance the performance of Automated Essay Scoring (AES) system.

### 2.3 Existing desktop tools in writing

At present, timing data captured by keystroke tools (i.e., desktop applications) can offer richer evidence to further contribute to writing research (Tian et al., 2023). Some existing studies have collected and analyzed log data, but failed to document either details about the design of the computer-based assessment, or details of how their programs capture students’ writing behaviors. These omissions occurred because their keystroke logging systems were developed by a company for commercial use (e.g., keystroke logging tool developed by Educational Testing Service, see details on Gong et al., 2022; Choi and Deane, 2021), or because of other copyright concerns, as the authors in question have explicitly stated (e.g., the tool developed by the authors that are Uto et al., 2020). These tools are not available for public users (e.g., practitioners or researchers) to use.

In contrast, Inputlog is available for public use. It has been widely applied in writing process research across a variety of settings. In K-12 contexts, it has been employed to investigate students’ developing writing skills (e.g., van Waes and Leijten, 2015). In higher education settings, Inputlog has been used to examine academic writing processes, particularly among second language (L2) learners, as well as to explore relationships between writing behaviors and text quality (Barkaoui, 2019). At the same time, it has also been employed in writing in workplaces professional communication (e.g., Leijten et al., 2014).

In terms of usage, Inputlog is a standalone desktop application that requires installation on individual computers prior to data collection (the user manual is available on https://www.inputlog.net/). Namely, researchers must first install the software and configure logging settings (e.g., sampling options, logging modes, and output formats). Writing tasks are then administered locally, often through applications such as Microsoft Word, where Inputlog runs in the background to record keystrokes, mouse movements, and other writing-related activities. The collected data can then be imported into Inputlog’s analysis modules or exported for further processing (e.g., statistical analysis). In all cases, this workflow involves manual steps for file transfer, organization, and preprocessing.

### 2.4 Clourite’s contribution to understanding writing process and writing instruction

The existing tools (e.g., Inputlog) provide a range of useful functionalities for studying writing processes. However, the cloud-based platform, Clourite, described in this article, may have the potential to deepen researchers’ understanding of writing processes and contribute to writing instruction. In particular, Clourite enables new research questions that are *difficult* or *impossible* to address using traditional desktop applications.

Consider the following research scenario. Bring-your-own-device (BYOD) has been widely adopted as a strategy to increase technology integration in classroom workflows and to give learners greater responsibility for using digital devices for educational purposes (Gabriel et al., 2022). In BYOD environments, students use their personal devices such as laptops, smartphones, and tablets, to support learning activities while developing both domain general skills and disciplinary knowledge. For example, Chou et al. (2017) conducted an experimental study with 46 junior high school students to examine the effects of the BYOD approach on language learning. Six quizzes were used as formative assessments during the experiment, together with a summative assessment, to evaluate students’ learning outcomes in English vocabulary and grammar. The results showed that the BYOD approach produced positive effects on students’ long-term transfer of learning. Students in the BYOD instruction group demonstrated steady improvement and performed better during the knowledge retention phase of the study.

Future research could extend this line of inquiry beyond grammar and vocabulary learning to investigate writing development in BYOD environments. Researchers could employ our cloud-based writing platform, Clourite, to examine whether BYOD-based instruction facilitates long-term transfer of learning in writing, that is, how writing skills evolve over time. Clourite enables the collection of richer writing data, including holistic writing scores provided by teachers over extended periods (e.g., a month, a semester, or an academic year), as well as writing process data captured through the keystroke logging system embedded in the platform. These data make it possible to construct a detailed writing profile for each student. For example, writing fluency indicators can be extracted from keystroke logging data to reflect students’ writing processes. As suggested in the literature, writing fluency refers to the degree to which transcription processes become partly automatized during writing. Previous research has shown that writing fluency is positively associated with writing quality; greater transcription fluency reduces the cognitive load associated with lower-level writing processes (e.g., transcription), thereby allowing writers to allocate more cognitive resources to higher-level processes such as idea generation and text organization (Gong et al., 2022). Such a detailed writing profile may also inform instruction by helping teachers guide students to focus more on idea generation and less on surface-level features.

Beyond the research questions described above, Clourite also enables studies that are *difficult* to conduct with traditional desktop keystroke logging tools. For example, a variety of statistical methods have been used to extract informative process features and examine the relationship between writing processes and writing products, many of which require large datasets. For instance, Sinharay et al. (2019) used data-mining techniques with a sample of more than 1,600 students, while He et al. (2025) employed deep learning approaches using 4,309 essays. In addition, large-scale naturalistic writing datasets collected in authentic class contexts are particularly valuable. This requires a writing platform that is minimally intrusive and can be easily integrated into everyday classroom workflows. Previous research has shown that perceived ease of use strongly influences whether teachers adopt digital tools in instructional practice (Granić, 2022). Tools that are easy to use reduce the technical burden on teachers and students, making it more feasible to incorporate digital platforms into regular instructional activities. Clourite is designed to support not only small-scale laboratory studies but also large-scale classroom-based data collection (e.g., across multiple school districts) in authentic educational contexts, such as daily learning activities (e.g., homework writing tasks) and summative assessments (e.g., final writing examinations).

The specific design details of the platform are presented in Section 3. The remainder of the article is organized as follows. Section 3 presents the architectural design of the writing platform and the details of the Bayesian model used to estimate parameters for each student. Section 4 reports the estimation results. Section 5 discusses the findings in relation to the existing literature.

## 3 Methods

In this section, the content is organized into four subsections. Firstly, we present how to use Clourite, that is, without downloading any software. It simply requires a few of mouse clicks for research users, teacher users, and student users. Secondly, we describe the modular architecture embedded in the design of Clourite, in which different functional elements are clearly encapsulated to capture the keystroke log data. Thirdly, we describe how large volume of raw information (e.g., keystroke log data) collected in Clourite are parsed (classified) into meaningful structured process data (e.g., between word pause linguistic context, within word pause linguistic context). Fourthly, with these meaningful and structured process data, we then proposed a Bayesian hierarchical mixture model to extract features. Lastly, we describe the context and the sample in the presented research.

### 3.1 Overview of the platform-Clourite

In Clourite design methodology, we deliberately ensure that multiple types of users can simultaneously access the website. Namely, researchers, teachers, and students can use the platform to perform different work seamlessly. In this section, we present the usage of Clourite with a few of screenshots (attached in Appendix A). Appendix Figure 1A is the screenshot for the *admin interface.* An admin person can sign in the website to create teachers accounts. For example, on this screenshot, this admin person input 8 teacher names in the blank, then, clicks “creates” blue button. The accounts for 8 teachers therefore have been created. The username for the first teacher is *disagra8teach1*, that indicates this is a teacher from school district *A*, teaching *grade 8*. Of course, this is one way to name teacher account, future users can create any handy names (e.g., TomSmith and JerryWang) they like.

Appendix Figure 2A shows the *teacher interface*. A teacher employs the account to sign in the website. The username and password are both *disagra8teach1*, that were created by the admin person previously. On the teacher interface, a teacher can create students accounts. For example, on this screenshot, the teacher *disagra8teach1* input 10 students’ ID numbers in the blank, then, clicks “create” blue button. The accounts for 10 students therefore have been created. Typically, a classroom teacher has a excel file at hand that contains all students ID numbers in the class. If that is the case, the teacher can simply copy the ID numbers column in the excel and paste onto the blank here. Based on the current capacity of our cloud server, we can accommodate around 2000 students accounts. In addition, Appendix Figure 3A shows that a teacher can also input a writing prompt and control time limits, then, the writing task therefore is ready to be delivered to students.

Appendix Figure 4A shows the *student interface*. A student employs the account created by the teacher previously to sign in the website. The student’s ID is 611 in teacher *disagra8teach1*’s class, then, this student’s username should be *disagra8teach1_611*, the password should be *611*. As soon as a student sign in the website, the timer starts working. The writing prompt is on the left, the typing area is on the right. The student can click “submit” button on the top left of the screen once finished writing.

Appendix Figure 5A shows the *teacher interface*. Once a student submits an essay, the teacher can see the whole essay text and watch replay. Namely, the teacher can watch how a student completed the essay from the very first letter to the very last letter. The fine-grained keystroke log system in Clourite enables the reconstruction of the whole essay. In addition, the teacher can input a holistic scoring result and click “submit” blue button.

Finally, Appendix Figure 6A shows the *admin interface*; the admin person manages multiple teachers accounts; the admin person can see every teacher’s interface including the scoring results provided by teachers, or watching replay of every student writing; the admin person can also be one of the teachers to perform the tasks such as creating writing prompts or grading essays. In addition, Appendix Figure 7A shows that the admin person can download all the structured writing process data (e.g., within word pause, between words pause), final score, student ID in a text file by simply clicking “retrieve data” blue button.

### 3.2 Design architecture of the cloud-based writing platform

**Figure 1:**
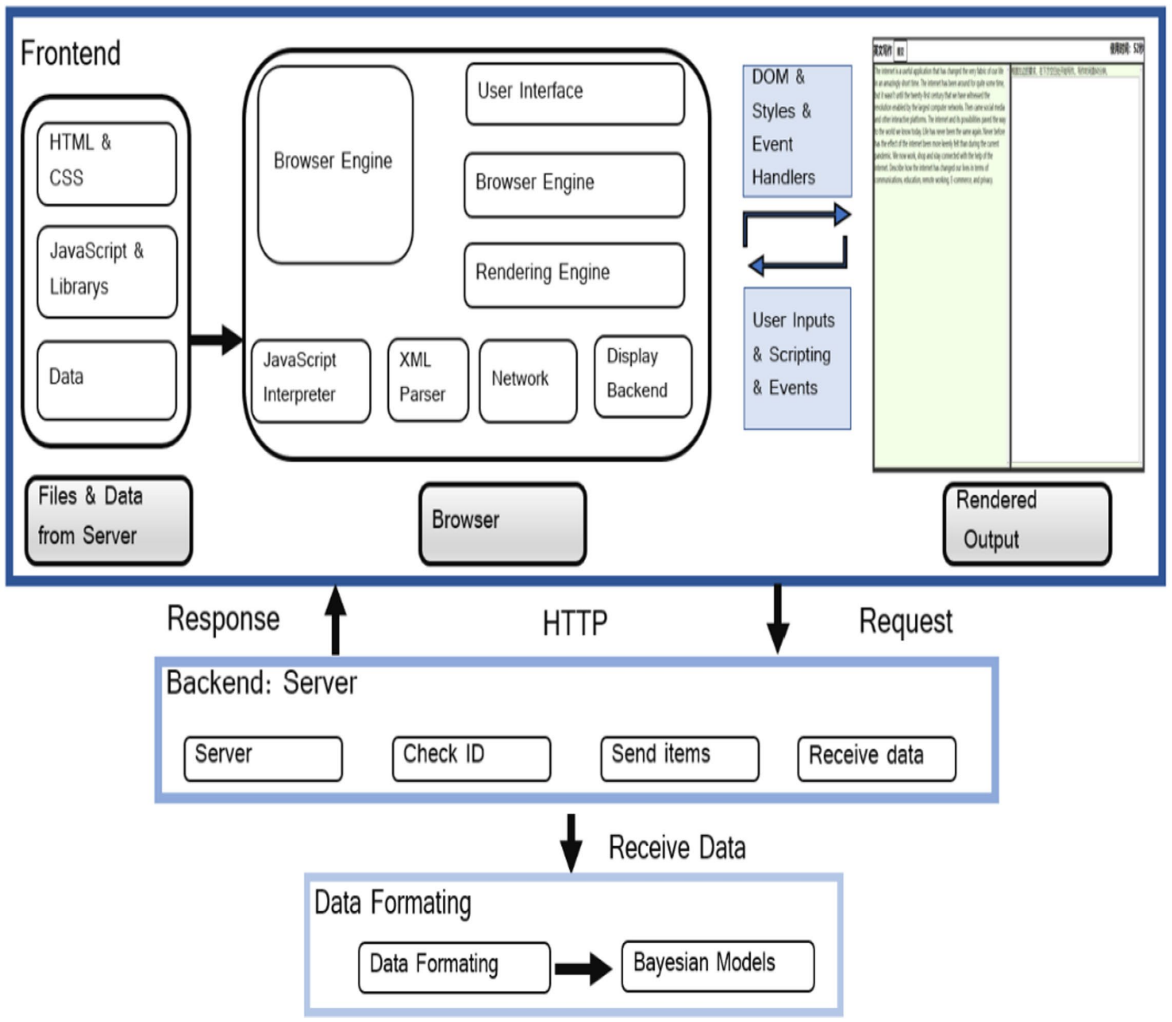
Writing platform-Clourite’s architecture.

A comprehensive and innovative cloud-based writing platform consists of the frontend and the backend. Figure 1 maps the design architecture for the present research. In the design component for the frontend, we (developers) created a website and the applications by using HTML/CSS and JavaScript. In the backend, we set up a server to receive data captured by the frontend. The design principle mainly contains three layers as stated in the following. Students’ typing behaviors can trigger the callback functions written with the JavaScript program, after which the program records a student’s behavior and converts the behavior into raw data. Subsequently, some items (i.e., questions, tasks, or writing prompts) in the platform are received from the server or raw data captured by the JavaScript program. They are temporarily stored in the buffer area of local computers; then, the raw data are sent to the server once students finish writing. After obtaining raw data, we use a parser to format the raw data. Finally, for the hardware, we use a Cloud Desktop that is sufficiently powerful to send items and receive data, such that a large number of students may write essays at the same time.

For the sake of formatting algorithms and reducing the server load, we distinguish tasks accordingly. The backend is in charge of the following: (1) working as the server of the platform, so that students can access the website; (2) sending writing prompts or items to students according to their student IDs; and (3) receiving raw data from students. Subsequently, we can structure the raw data into formatted data, which includes information for further computations. The purpose of formatting the data is to import them for later analysis. The data also contain other information (e.g., student ID). Then, the data are sent to the cloud server that converts the data to the instances of the class variable, and reconstructs how exactly the student has completed the essay.

### 3.3 Pause event classification

**Figure 2:**
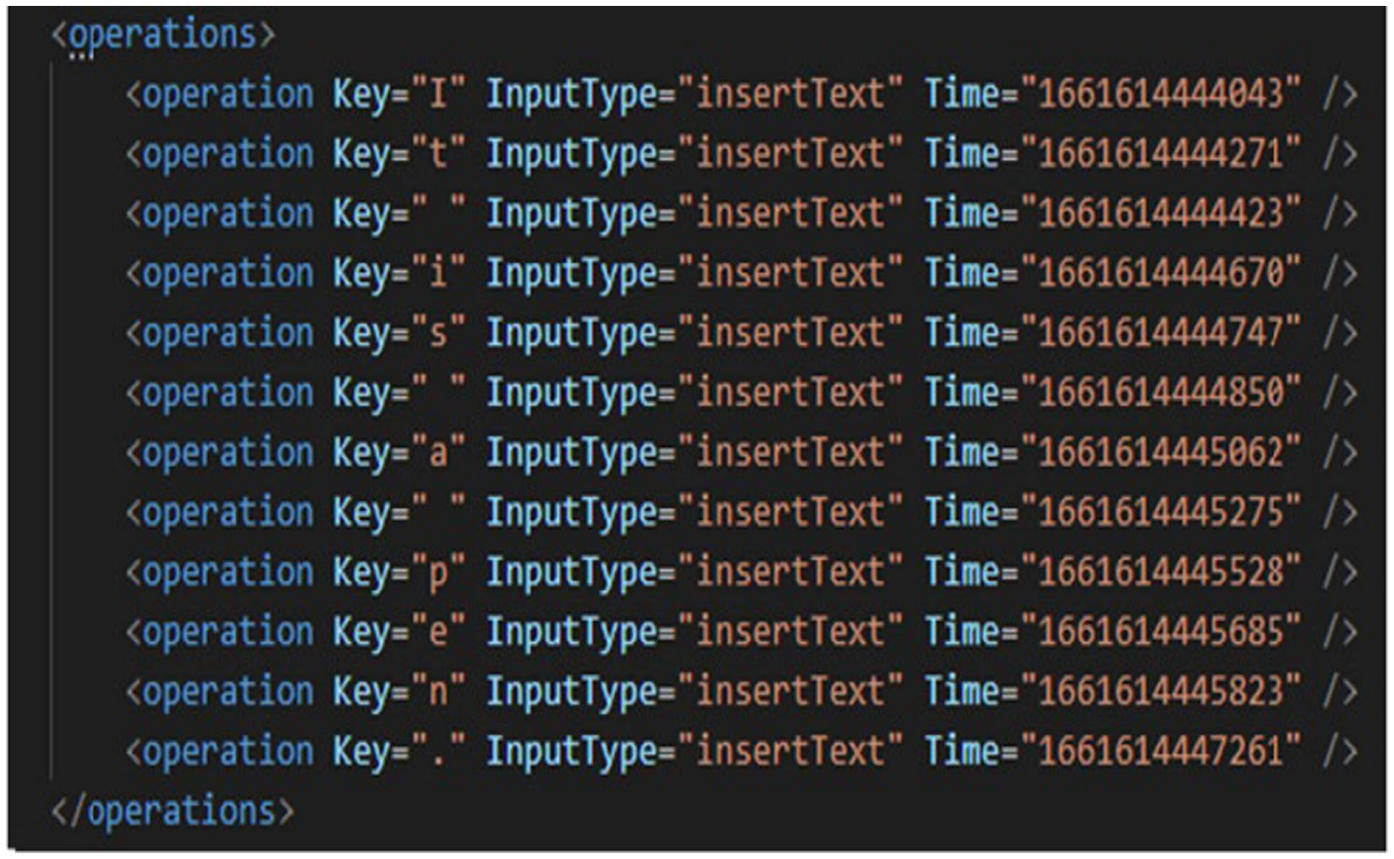
Typing behavior reflected in keystroke log.

The platform, Clourite, can automatically parse (classify) the raw data into various structured dataset (e.g., *within word* pause, or *between words* pause). The structured data can then be downloaded immediately by the researchers to conduct further statistical analysis. A *between words* pause is identified when a pause occurs around the pressing of the space bar; a *within word* pause was defined as the time interval between two consecutive keystrokes, measured in milliseconds. This definition is consistent with the literature (e.g., Zhang and Deane, 2015; Guo et al., 2018). Figure 2 shows a simple example of a log of keystroke activities when typing a sentence, such as “It is a pen.” The time stamps allow us to compute the length of the pause (in milliseconds), according to the linguistic context. By classifying the operations along the order of their time stamps, we can convert the data from instances of a key-based variable to instances of a word-based variable, which means we know how the student typed each letter.

Taking the word “pen” as an example, the values of keys in the variable are “p,” “e,” and “n.” Each instance of variable has its own time stamp. The application makes them into one, as “pen,” and combines their information, thereby creating attributes “pen,” and “Operation Type” for this. We can reconstruct the whole essay and map the time stamp to each character of the essay. Consequently, we can compute various writing behaviors such as the pauses between words and the pauses within a word. In addition, a punctuation mark (e.g., comma, period, semicolon, or question mark) is treated as *between sentences* pauses. With the information of “Input Type,” “Key” and “Time,” the formatting script can simulate the process of a student’s writing. For example, for the *between words* linguistic context, taking “a pen” as an example. $\mathrm{\Delta t}=t_{2}-t_{1}$ , where $t_{1}$ is the time stamp of “a” and $t_{2}$ is the time stamp of “p” in “pen.”

### 3.4 Bayesian hierarchical mixture model

Finite mixture models are used when the observation events are drawn from two or more different subpopulations (Biernacki et al., 2000). Each subpopulation is termed as a *mixture component* in the model. A random variable comes from a population with *K* components. The notation *k* represents the mixture component, which adopts a value between 1 and *K*. Equation 1 probability density function of the mixture model with *K* components for a random variable $\text{of}\;Y_{n}(n=1,\cdots ,N)\;$ is given by:

$$f(y_{n}|\theta )=\sum_{k=1}^{K}\pi_{k}f_{k}(y_{n}|\theta_{k})$$ (1)

Usually, mixture components are assumed to follow the same parametric family with different parameter vectors. The *mixing proportion* parameter is $\pi_{k}$ , that is the proportion of the population from *k*. The constraint of $\pi_{k}$ is $\sum_{k=1}^{K}\pi_{k}=1$ . In practice, the component membership typically is unknown. In the present research, the unobserved variable Z is termed a *latent mixture indicator*. Thus, a set of latent indicator variables $Z_{\mathrm{nk}}$ , where $Z_{\mathrm{nk}}$ takes the value 1 if observed pause *n* belongs to component k , and is zero otherwise.

As introduced in the Section 2 above, cognitive models have posited theoretical rationales about the writing process. Subsequently, the assumptions made in this present research include: (1) writing involves multiple processes operating simultaneously; (2) any observed pause event can be drawn from one of the latent cognitive processes; (3) The mixture components in the mixture model correspond one-to-one to these latent cognitive processes.; (4) a longer pause comes from higher level cognitive processes, whereas a shorter pause is drawn from lower-level cognitive processes. In addition, the mixture model is not identifiable. So, the researchers need to define the latent mixture component (McNicholas, 2016). The traditional approach to determine the number of components is trying for small values of *k* (i.e., *k =* 2, 3, …, *K*). Our previous study (i.e., (Li, 2021)) found that the two-component mixture model seemed sufficient to fit the keystroke log data for both *within word* linguistic context and *between words* linguistic context. In the literature, two mixture components can be termed as “Pause Component” and “Longer Pause Component”, as suggested by Deane and Zhang (2020). Pause Component indicates the mixture component with the smaller mean. Longer Pause Component indicates the mixture component with the larger mean.

Consistent with prior research (e.g., Guo et al., 2018; Gong et al., 2022), pause durations in writing are assumed to reflect different levels of cognitive process. Specifically, shorter pauses are generally associated with lower-level cognitive processes involved in transcription, such as typing, spelling, and lexical access. In contrast, longer pauses are thought to reflect higher-level cognitive processes, including text planning, deliberation, and evaluation of the text produced so far. Accordingly, in the present study, Pause and Longer Pause are treated as observed indicators that inform the estimation of two latent constructs: lower-level cognitive process and higher-level cognitive process.

**Figure 3:**
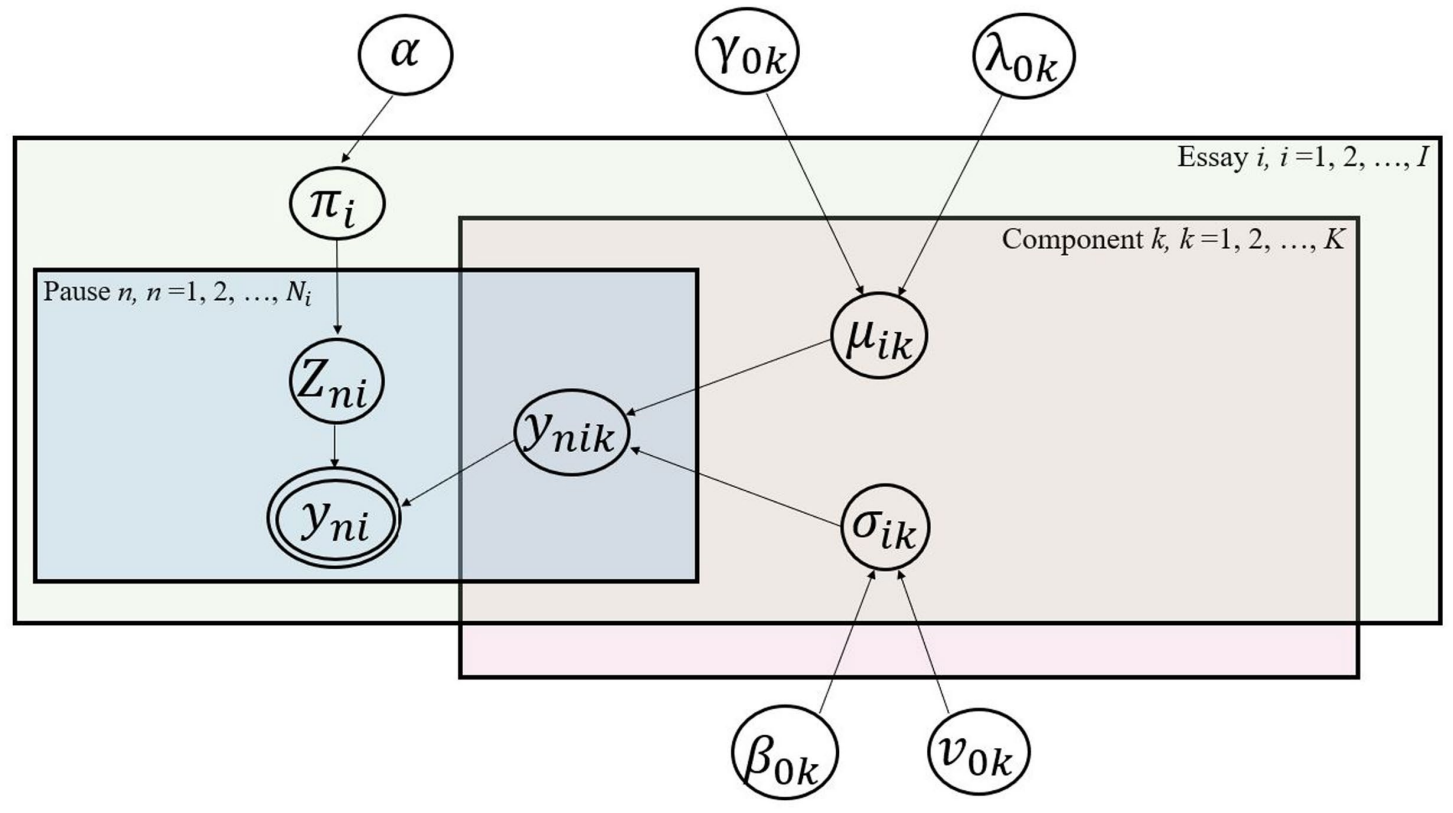
Notation of the proposed Bayesian odel.

Based on the mixture model described above, in the present research, we add more one layer onto the mixture model, and estimated the parameters in the fully Bayesian framework. Consequently, the proposed model in this research is the two-component Bayesian hierarchical mixture of model. The model is illustrated in the plate notation in Figure 3. In addition, we chose to focus the data from *within word* linguistic context. In the proposed model, we denote each essay *i, i* = 1, 2, 3, …, *I*. Pause event *n* is nested in each essay. That means, Level 1 is the *within essay* level. Level 2 is the *across essay* level. Thus, this adds the Level 2 parameters (see Equations 2-4), that is, $\gamma_{0k}$ , $\lambda_{0k}$ , $\beta_{0k}$ , $v_{0k}$ .

$$\pi_{i}=(\pi_{i1,},\ldots ,\pi_{\mathrm{ik}})\sim \text{Dirichlet}\;(\alpha_{1},\ldots \alpha_{k})\;\pi_{i}$$ (2)

$$\mu_{\mathrm{ik}}\sim N(\gamma_{0k},\lambda_{0k})$$ (3)

$$\log(\sigma_{\mathrm{ik}})\sim N(\log(\beta_{0k}),\nu_{0k})$$ (4)

Let *δ* the complete parameter ( $\pi_{i}$ , $\mu_{\mathrm{ik}}$ , $\sigma_{\mathrm{ik}}$ , α , $\gamma_{0k}$ , $\lambda_{0k}$ , $\beta_{0k}$ , $v_{0k}$ ). The likelihood for an essay becomes:

$$L(Y|\delta )=\sum_{i=1}^{I}\log L_{i}(Y_{i}|\pi_{i},\mu_{\mathrm{ik}},\sigma_{\mathrm{ik}})$$ (5)

The foregoing philosophical approach to the model setup in the fully Bayesian framework contains a series of steps. The first step is probability specification. The models typically are often very large (e.g., large number of parameters or complex probability specifications). In this research, each essay includes 5 parameters: two means, two variances, and one mixing proportion, as shown in Figure 3. The second step is the estimation of the parameters using Markov Chain Monte Carlo (MCMC) algorithms. The third step is to summarize the posterior distribution by checking the model convergence. The *convergence* simply indicates that the MCMC samplers converged to the stationary distribution to obtain the samples. The model convergence is reported in the section below.

### 3.5 Participants and data collection

The data were collected at a vocational college in China from two cohorts of students enrolled in a general English course in the Fall semesters of 2024 and 2025, respectively, both taught by the same instructor. The writing task was assigned as an optional extra-credit activity. In the Chinese EFL context, College English is a compulsory course for non-English majors and is also widely offered in vocational colleges. Its instruction is guided by the College English Teaching Guidelines (Ministry of Education, 2020), which emphasize language proficiency, academic literacy, and critical thinking. Within this framework, argumentative writing plays an important role, as it requires students to construct and justify positions with evidence. Students completed an opinion-based argumentative writing task based on a social topic concerning “The Internet and Our Life”. The writing prompt is presented to the students as the following:

*In 2018, the* “dream of becoming an internet celebrity among young people” *became a major social topic. The data below are taken from the City College Graduates Employment Intention Survey Report. Please discuss your views on this issue.*

*The top three most popular career choices were: live streamer (25.12%), internet celebrity (19.06%), and new media operator (18.2%).*

Students were required to express and justify their views in English. The writing tasks were completed during class using students’ own devices via the Clourite platform. The time limit was 30 min. Once students signed into the system, the timer started automatically; when time expired, the session closed and all data (text and keystroke process data) were saved. Students could also submit their essays by clicking the “submit” button on the interface. No other questionnaires were used. The independent review was exempted, as this writing task was embedded within the optional writing practice offered during regular instruction.

Essays were scored independently by two doctoral students in English using a holistic rubric focusing on (1) argument development and support, and (2) coherence and cohesion. Each essay was rated on a scale from 0 to 5 (in 1 increments). Inter-rater reliability, measured by Krippendorff’s alpha (Hayes and Krippendorff, 2007), was 0.92, indicating high agreement. The two scores were summed to yield a final score ranging from 0 to 10. The final scores are the human scores which are used as writing product measure and employed to identify the association between writing process features and writing product measure (see the next section below). In addition, essays with a score of 0 (e.g., empty responses or writing durations under 2 min) were excluded. A total of 309 students participated in the study (169 female and 140 male), with ages ranging from 18 to 19. This sample size is comparable to prior studies on writing process analysis (e.g., Uto et al., 2020; Conijn et al., 2022). In total, 12 essays (approximately 4%) were removed, resulting in a final sample of 297 essays.

## 4 Results

### 4.1 Descriptive statistics

**Figure 4:**
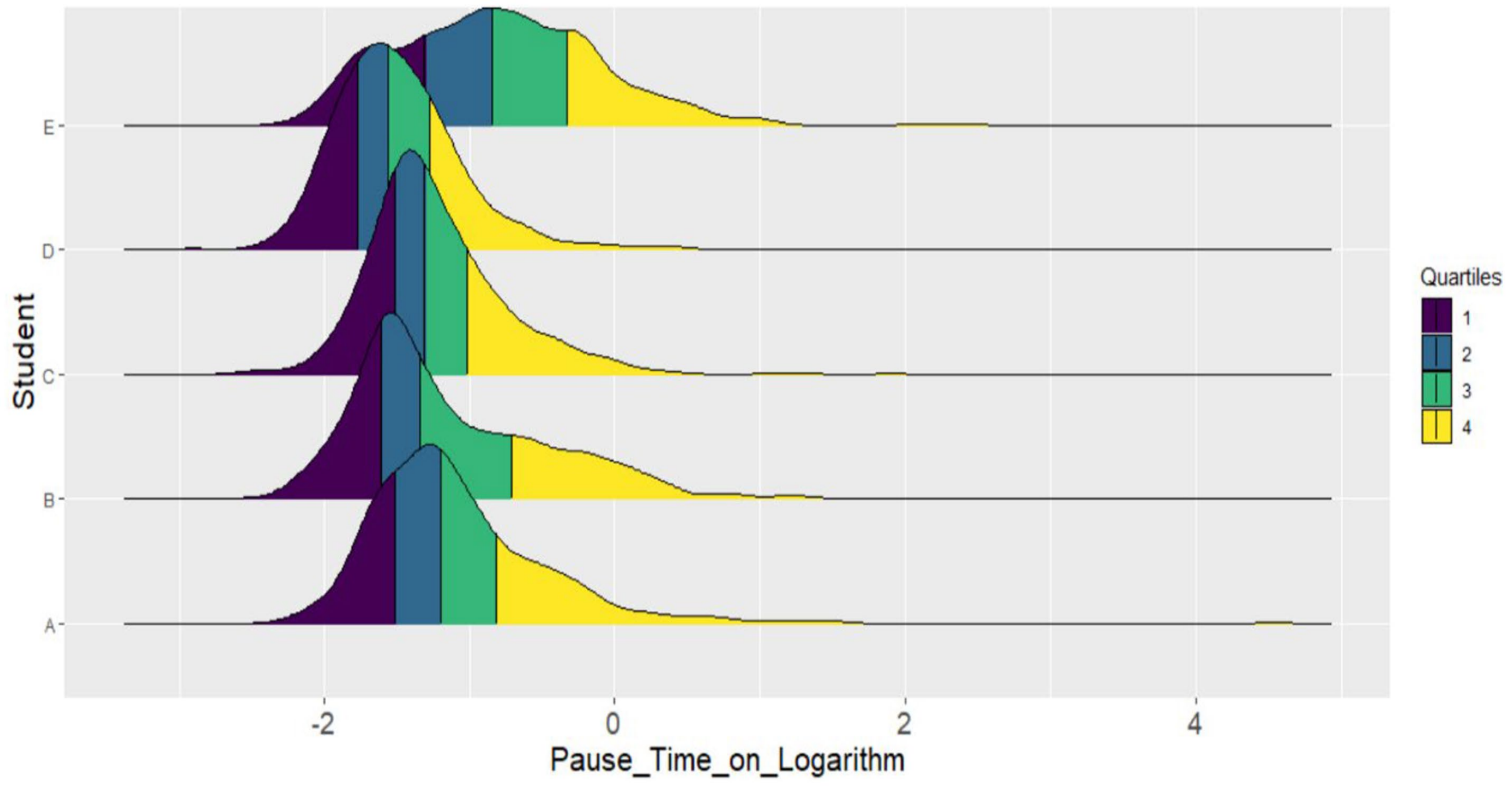
Pause data across five students.

In a body of work (see details on Zhang and Deane, 2015; Guo et al., 2018), *within word* pause was defined explicitly as the time interval between two consecutive keystrokes measured in milliseconds. In the present research, we endorsed this definition. In these authors’ work, pause times are reported in seconds, in addition, statisticians typically tend to apply to logarithm transformation to reduce the skewness of time data. This is also the case in the present research. Namely, most essays possessed heavy tails (high kurtosis) and were skewed even after we transformed them onto the logarithmic scale. For illustration purposes, we randomly chose 5 students to show the distributions on the logarithmic scale (see Figure 4). The X-axis is the pause time (measured in second and on logarithmic scale), whereas the *Y*-axis is the density for these 5 students.

### 4.2 MCMC convergence diagnostics

As described above, we employed a Bayesian hierarchical mixture model to extract process features. In this section, we briefly outline the parameter estimation procedure, through which five parameters are estimated and subsequently used as features. For the model estimation, we employ a set of MCMC diagnostics tools to present chain convergence results. If the model has converged, the samples from the conditional distributions can be used to summarize the posterior distribution of parameters, in this case, the mean, variance, and the mixing proportion for each essay. (The specific code used is shown in the Appendix B attached to this article). The Gibbs samplers are implemented in a JAGS program (Plummer, 2003) to estimate the parameters. A JAGS program is often used in the R program environment (R Core Team, 2022). We used the R2jags package (Su and Yajima, 2022) that was deigned to call JAGS programs in R. The package also has a built-in monitoring tool for assessing chains convergence.

For the convergence, we mainly analyze the following aspects. Firstly, we checked the R-hat. The R-hat ( $\hat{R}$ ) is the potential scale reduction factor (PSRF, Gelman and Rubin, 1992), that has been used often in assessing the chains convergence. It is a numerical value used when two or more chains are in the estimation, in order to reflect the convergence summary that reflects the ratio of within- to between- chain variance. If the chains mixed well, the $\hat{R}$ value should be around 1.

**Figure 5:**
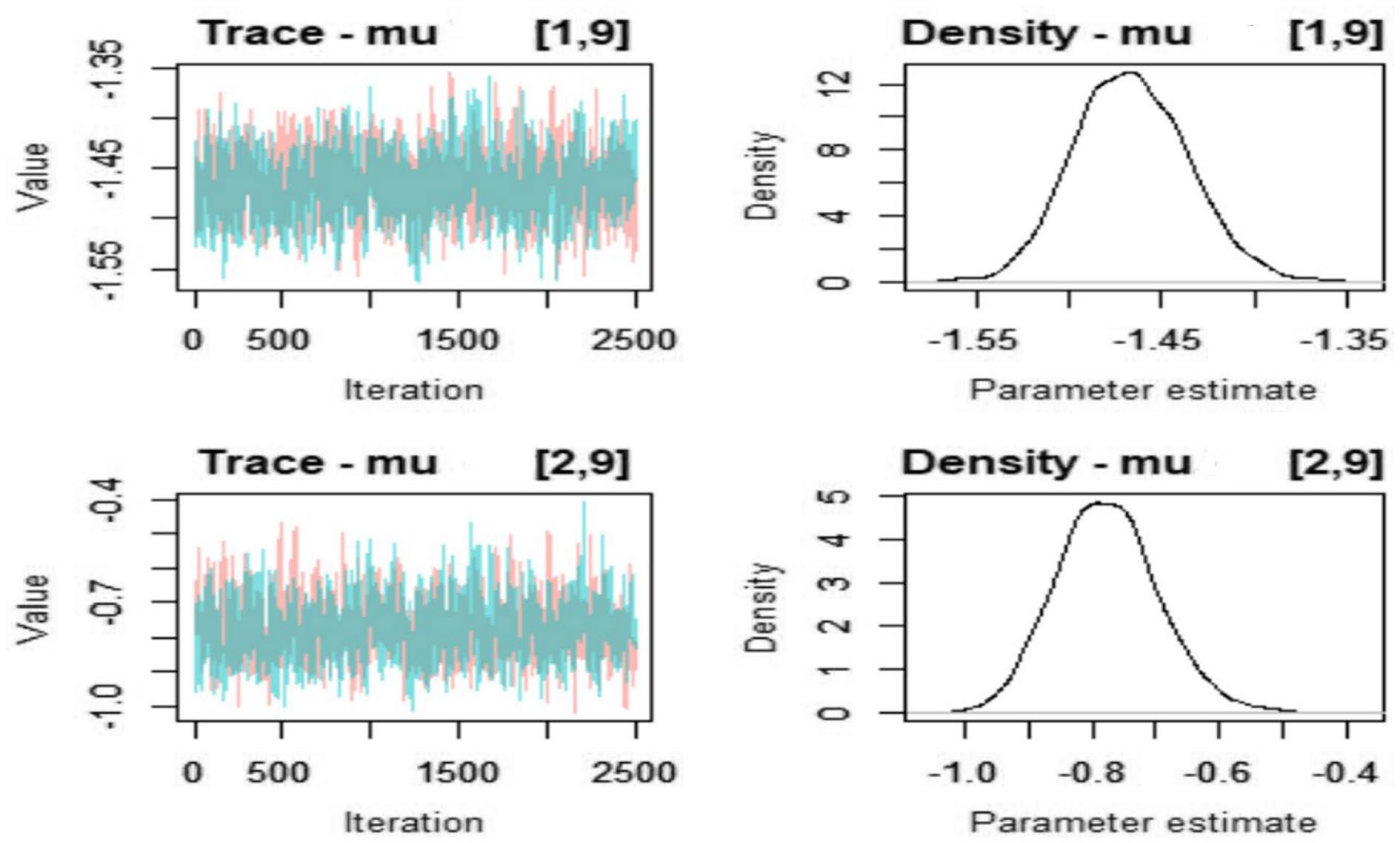
Traceplots and kernel density plot of a mean parameter for a student.

Secondly, we checked the trace plot. The trace plot is graphical representation for the parameter value at each iteration. It reflects sampling behavior and evaluates mixing across chains and convergence. For the convergence, the trace plot should be close to the mode of the stationary distribution with minimal fluctuations. Moreover, if the chains are converged, the posterior distributions of the parameters should look unimodal and smooth. In this research, the trace plots and the kernel density of the posterior distributions look desirable in terms of the chains’ convergence. These indicate that Markov chains converges to the stationary distribution. For illustration purposes, Figure 5 depicts the traceplot and kernel density plot for the mean parameter of a component, for Student ID 009.

Thirdly, we checked the autocorrelation. The degree of autocorrelation is an important index in the MCMC convergence diagnostics, which determines the degree of dependence between the draws of a chain. The simulated value of *θ* at the (*t* + 1) iteration is dependent on the simulated value at the *t* iteration. A high correlation between successive iterates prevents the algorithm from exploring the entire region of the parameter space. In this research, no high autocorrelation is found across parameters.

### 4.3 Correlation between process feature and writing product measure

**Figure 6:**
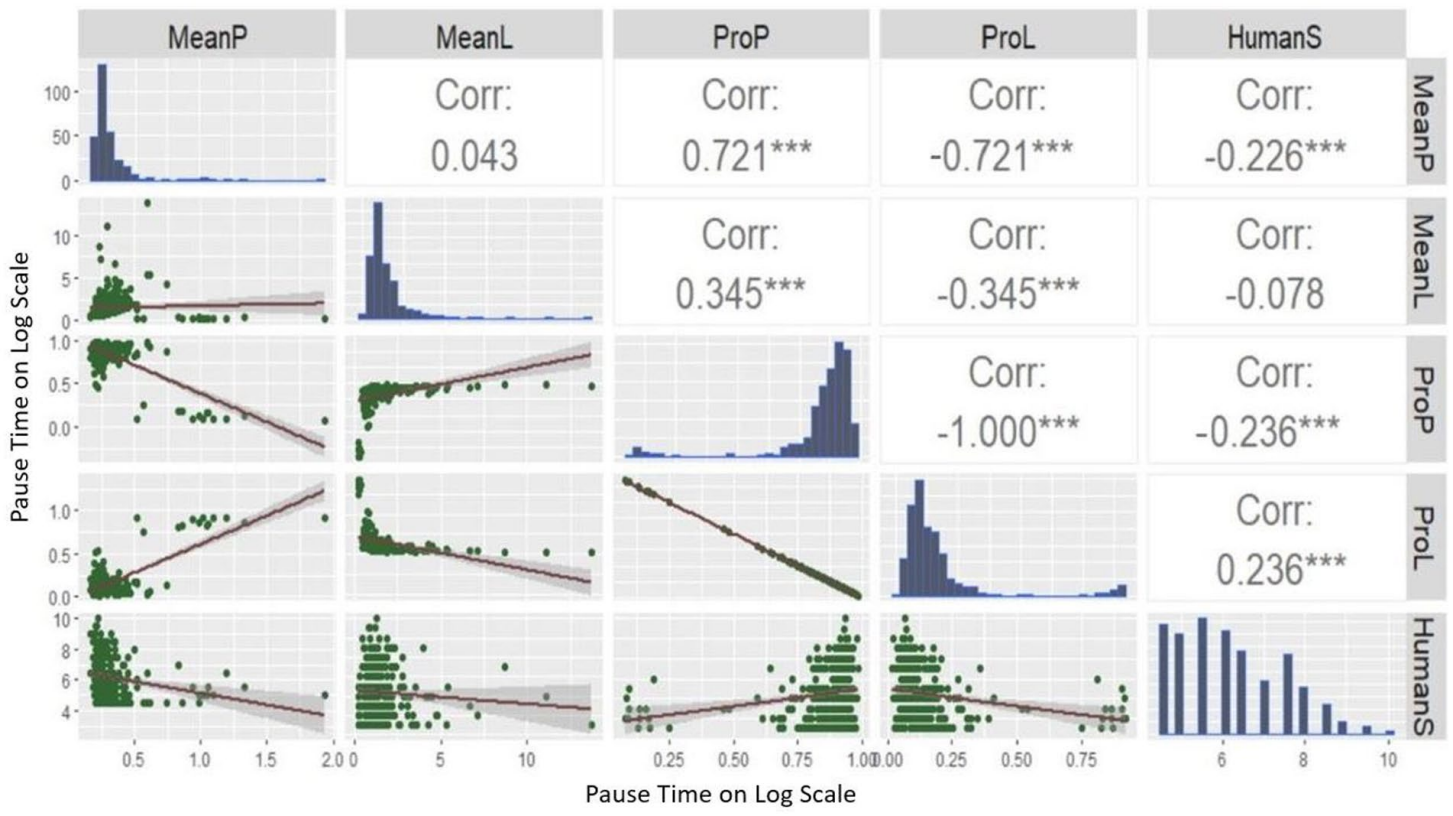
Correlations between process features and product measure. *p* < 0.01, *p* < 0.001. HumanS is the product measure provided by the human raters; MeanP is the mean parameter in the pause component; MeanL is the mean parameter in the longer pause component; ProP is the mixing proportion parameter in the pause component; ProL is the mixing proportion parameter in the longer pause component.

In the previous section, the MCMC diagnostic output analysis showed that the chains’ convergence seemed favorable. Subsequently, for each parameter, we used the mean of the posterior distribution as the estimated quantity. The five mixture parameters are used as the relevant writing process features. In this section, we examined the relationship between process features and product measure. Specifically, we computed the correlations among a series of variables reflecting (1) the extracted process features and (2) human scores. Human scores reflected students’ writing competency, as well the quality of each final writing product, or the holistic score provided by the human raters. The extracted process features include: the estimated mean, variance, and the mixing proportion for each essay in the two-component Bayesian hierarchical mixture model. The correlation plot among these variables is shown in Figure 6.

As shown the magnitudes of the correlation coefficients in Figure 6, the longer duration a student typically stayed at the surface characteristics, the lower human score the student received. Namely, the mean of Pause Component has a significantly negative relationship with human score (*r* = −0.226). In addition, that students who are able to use these basic processes fluently may allocate more cognitive resources to the higher-level processes, such as idea generation, coherence, or sentence planning. Namely, the smaller magnitude on the mean of Pause Component is significantly associated with the larger number of pause behaviors in Longer Pause Component (*r* = −0.721).

Finally, the correlation pattern is the tentative way to profile students from the perspective of the overall writing process; that is, the pattern may identify students with high and low writing competency. As shown in the plot, the mixing proportion parameters are not homogeneously spread out. Students with lower human scores have a wider scope of values on the mixing proportion parameter, whereas students with higher scores do not possess this trend.

## 5 Discussion and conclusion

In this section, we discuss our research results in the connection to the existing literature. Firstly, we found that Clourite has desirable functionalities as designed. Clourite is designed to support the automatic collection and classification of writing process data in authentic educational contexts. Unlike traditional desktop keystroke logging tools, Clourite integrates writing task administration and process data collection within a unified environment. Its cloud-based architecture enables centralized data management and cross-device compatibility in educational settings. In addition, the platform enables users to watch replay, supports the automatic recording of writing sessions, and download the dataset consisting both process data and product data.

In sum, modern technology has offered a variety of methods to gain the empirical evidence of writing processes. Compared with wearable electronic devices such as eye-tracking tools, a cloud-based platform is considered a much more non-intrusive mode of capturing individuals’ process data. To reshape the future digital writing community, we have made a small step forward by developing a cloud-based writing platform, Clourite, from which a broad range of users may benefit.

Secondly, in terms of quantifying the empirical keystroke log data, we chose to model the pause events in the *within word* category. In the literature, a series of writing behaviors has been identified (Almond et al., 2012); for example, *jump editing* occurs whenever a student moves further away from the current word to a new location for editing purposes. But this sort of behaviors was comparatively rare (e.g., occurring an average of 6 times per essay), thus making them difficult to model (Guo et al., 2020). Consequently, researchers tend to use non-parametric methods (i.e., exploratory cluster analysis) to classify them (e.g., Zhu and Gu, 2023).

In contrast, for the parametric methods, when the number of observations is small, but the number of parameters is large in a model, the estimation may not achieve the desirable statistical stability. Thus, in this research, we chose to focus on pause events that occur often during writing, such as observations in the *within word* category. We used a two-component Bayesian hierarchical mixture model to analyze the data. The mixture parameters extracted from the model were treated as the *writing process features*, whereas the holistic score provided by human raters was treated as *product measure* representing writing performance.

Thirdly, the existing literature typically adopt two modeling procedures. One modeling procedure is that making assumptions based on cognitive models, then, mapping the statistical results onto the cognitive structure. For example, a series of studies (Guo et al., 2018; Gong et al., 2022) assume that pauses within word should partly reflect lower-level cognitive processes (e.g., transcription skill, spelling difficulty). In contrast, the other modeling procedure is that researchers do not make the assumptions, as Conijn et al. (2022) stated. Researchers use data driven approach to identify what features are the most useful to predict writing quality (human scores). Simply put, imagine that a student paused 10 s when he was spelling “pen.” This does not reflect he had major cognitive difficulty on spelling. This happens simply because he stretched; or he was distracted by the sound of a bird outside the window (see details on Tian and Cushing, 2025).

In the present research, we used the former modeling procedure. Namely, students who can perform lower-level processes fluently are better able to allocate cognitive resources to higher-level activities, such as idea generation, coherence, or sentence planning (see Figure 6). Also, the mixing proportion parameter emerged as an informative process feature. The results indicate a non-uniform relationship between this parameter and writing performance (as measured by human scores), with a stronger association observed at the upper end of the distribution (as shown in Figure 6). Specifically, weaker writers exhibited greater variability in the mixing proportion parameter, whereas stronger writers showed a more restricted range. This pattern suggests that more frequent short pauses are associated with lower writing performance. Frequent pauses within words may reflect difficulties in spelling or lexical retrieval, rather than engagement in higher-level cognitive processes.

In addition, the results raise relevant questions regarding the extent to which the properties of the extracted features and their interpretations may vary across different settings. Writing settings can differ along several aspects, including (a) the complexity of prompts, (b) the duration of writing tasks, (c) genre, and (d) characteristics of the writer population (e.g., L1 vs. L2 writers). As suggested in the literature, establishing feature consistency is an initial first step (Guo et al., 2018), where consistency refers to the extent to which feature properties behave similarly across settings. For example, Guo et al. (2019) examined feature consistency across writing genres and found that the extracted features exhibited similar patterns in both policy recommendation and argumentative writing tasks. Deane et al. (2018) examined whether features vary by item type (i.e., writing tasks) and found that they were consistent across exemplary short-answer responses and single-session, single-draft essay-writing tasks.

It is recognized that participants’ characteristics are a systematic source of variance in language performance (Sok et al., 2021). For example, prior research (e.g., Choi and Deane, 2021) has examined whether L2 writing processes resemble those of L1 writers and whether findings from L1 contexts can be generalized to L2 settings. Contributing to this line of inquiry, the results of the present study are consistent with those reported in our previous work (see details in (Li, 2021)), which examined middle school students whose first language was English. In contrast, the current study focuses on college students whose first language is Chinese. By comparing features across these two samples (L1 vs. L2 writers), we find that the extracted process features are largely consistent. In particular, the mixing proportion parameter emerges as a stable feature. There is a non-uniform relationship between pause time and human scores: students with lower scores exhibit a wider range of values in the mixing proportion parameter, whereas higher-scoring students do not display this pattern.

## Data Availability

The original contributions presented in the study are included in the article/Supplementary material, further Inquiries can be directed to the corresponding author/s.
