# Supplementary material for: A Bayesian hierarchical mixture model for extracting process features in a newly developed cloud-based writing platform
Source: Front Psychol. 2026 May 28;17:1792834. doi: 10.3389/fpsyg.2026.1792834 (PMC13254725; doi:10.3389/fpsyg.2026.1792834)

**Appendix A**

**The Usage of Clourite with Screenshots**


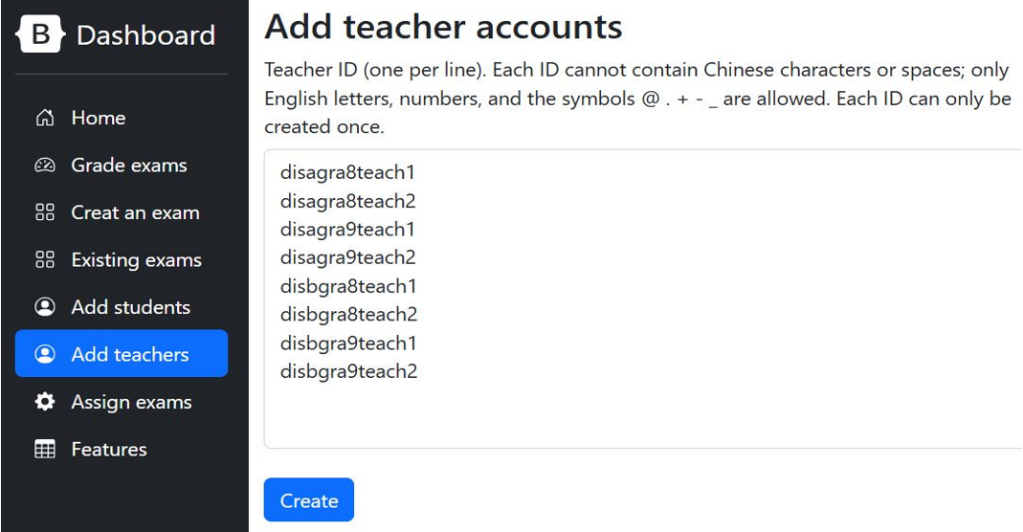


**Figure 1a**

*The Admin interface in Clourite—Create Teacher Account*


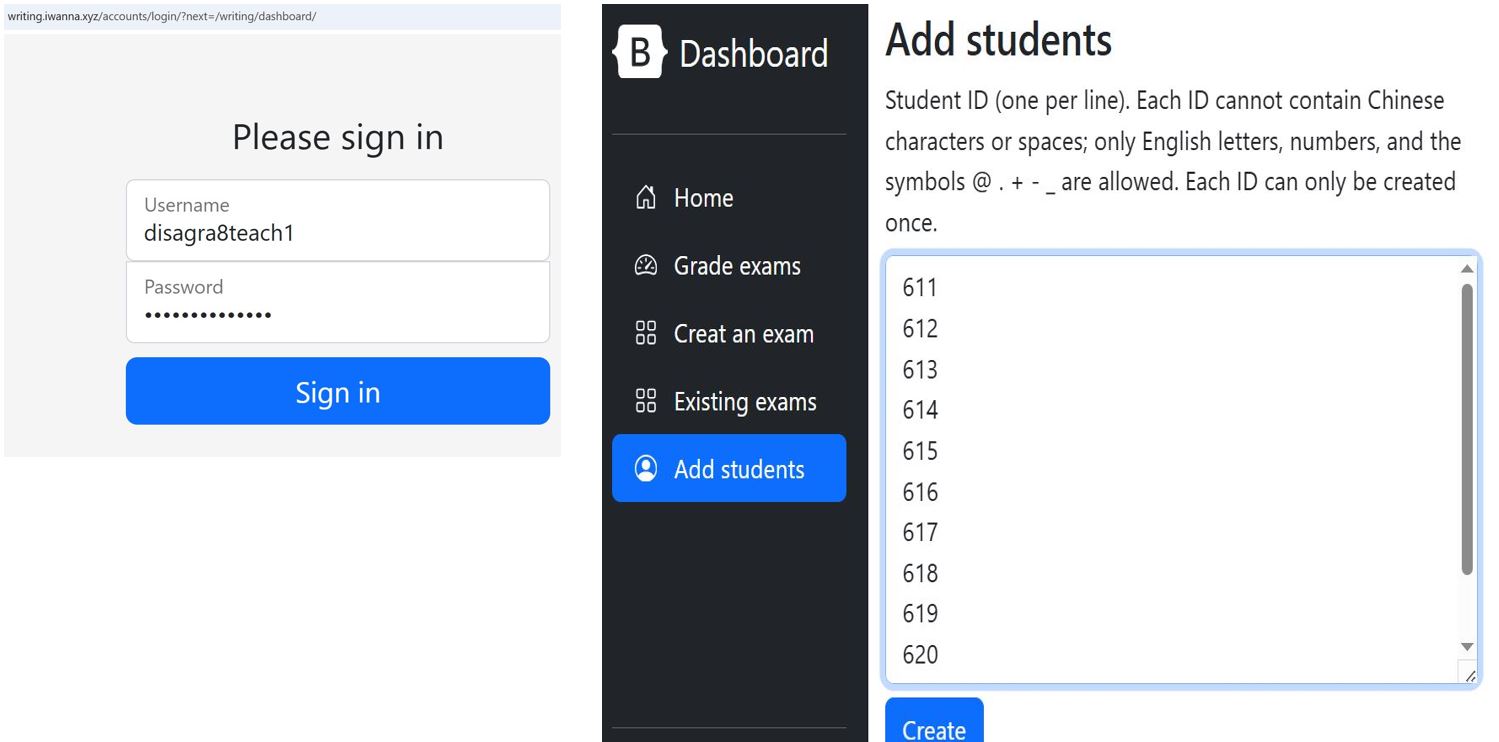


**Figure 2a**

*The Teacher interface in Clourite-Create Students Accounts*


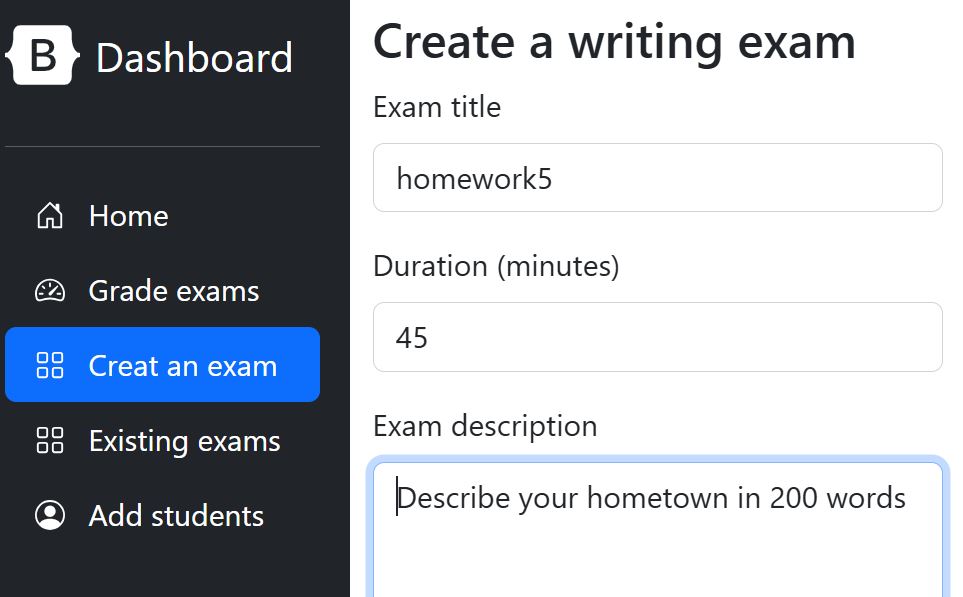


**Figure 3a**

*The Teacher interface in Clourite-Creating Writing Assignment* (*Prompt*)


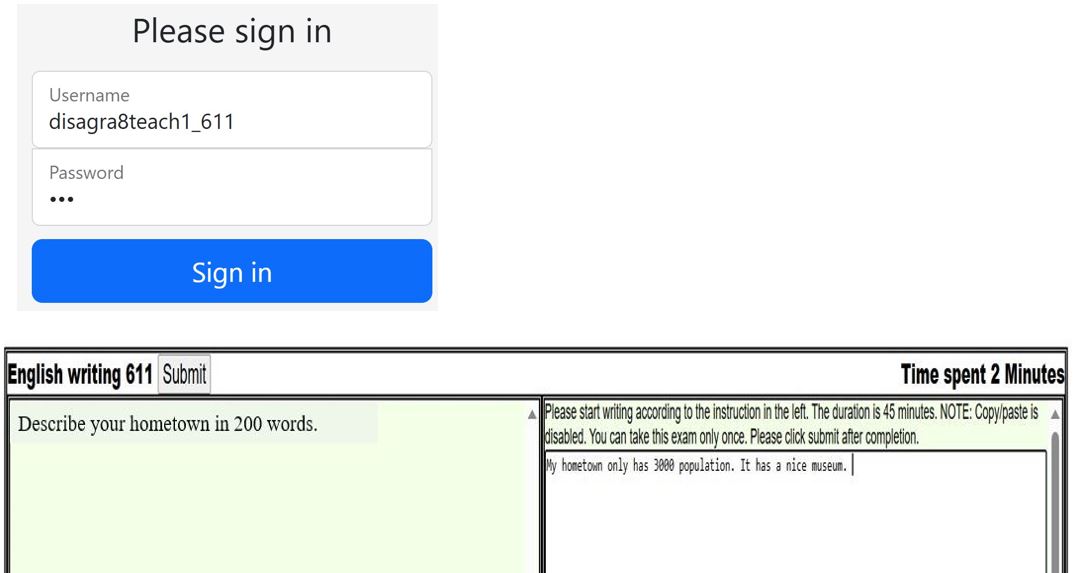


**Figure 4a**

*The Student interface in Clourite*


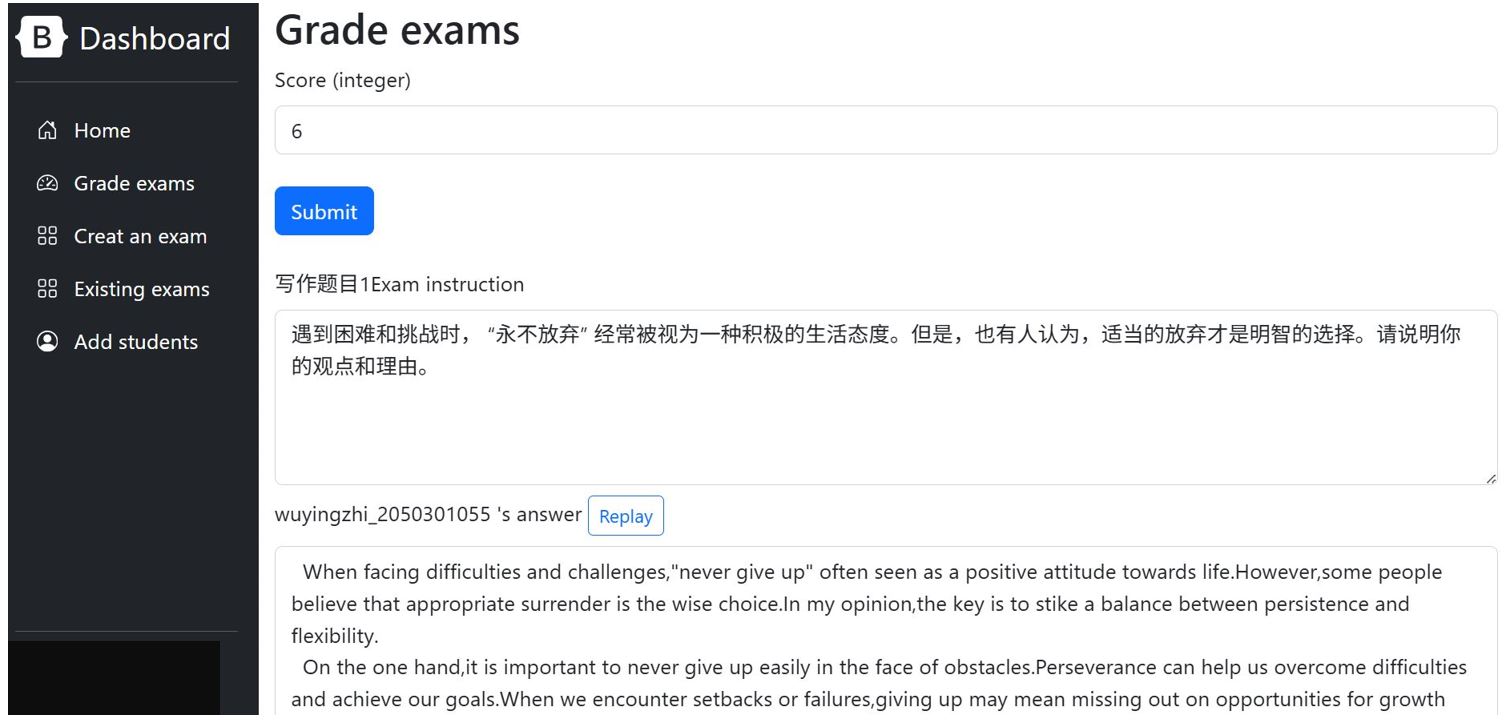


**Figure 5a**

*The Teacher interface in Clourite-Grade Essays*


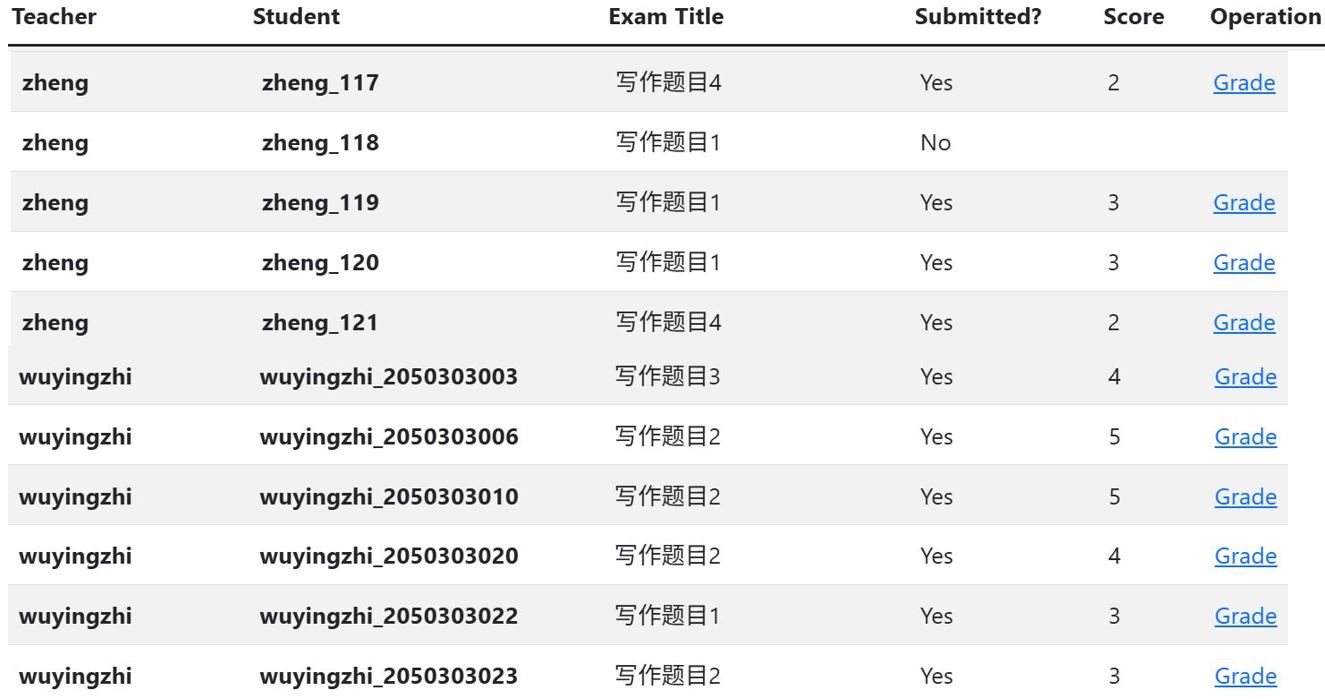


**Figure 6a**

*The Admin interface in Clourite-Check All Data*


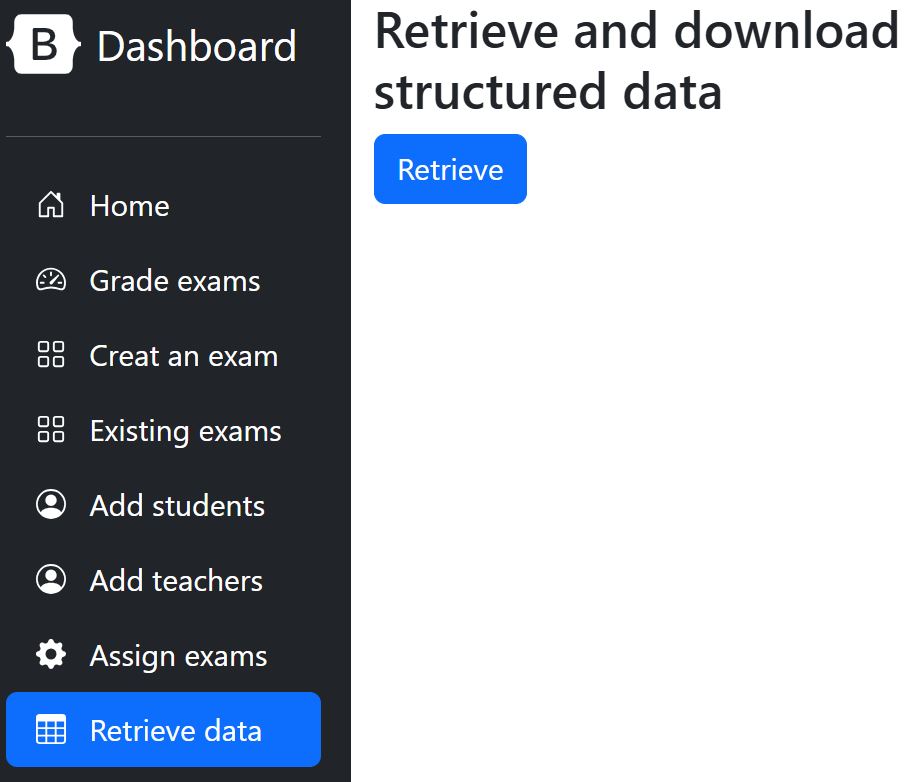


***Figure 7a***

*The Admin interface in Clourite-Download Process Data*

**Appendix B**

**JAGS code and R code**


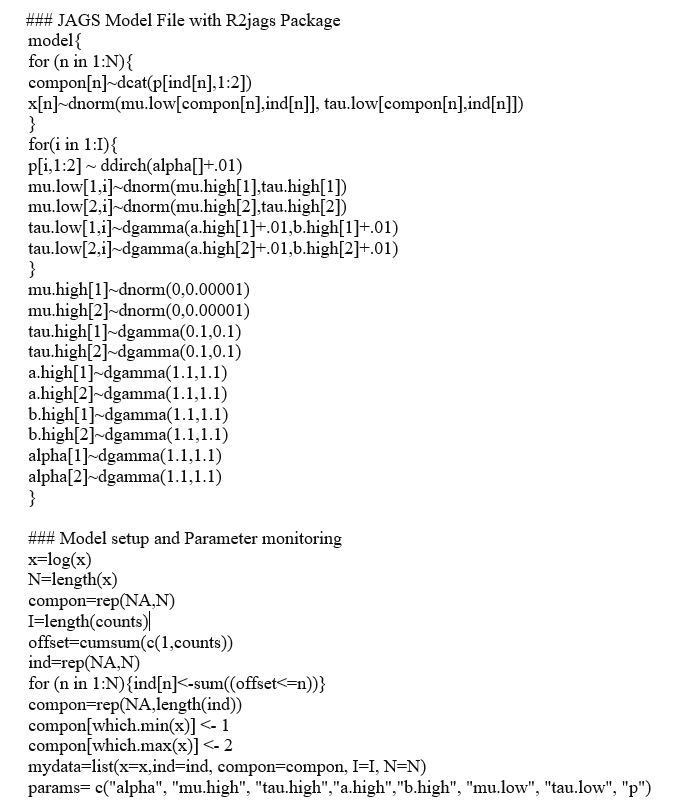

Supplement: Supplementary file 1 [file Supplementary_file_1.docx]
